# Set Packing Optimization by Evolutionary Algorithms with Theoretical Guarantees

**DOI:** 10.3390/biomimetics9100586

**Published:** 2024-09-27

**Authors:** Youzhen Jin, Xiaoyun Xia, Zijia Wang, Xue Peng, Jun Zhang, Weizhi Liao

**Affiliations:** 1 School of Information Science and Engineering, Jiaxing University, Jiaxing 314001, China; jinritian521@sina.com (Y.J.); liaowz@zjxu.edu.cn (W.L.); 2 School of Computer Science and Cyber Engineering, Guangzhou University, Guangzhou 510006, China; 3 School of Mathematics and Systems Science, Guangdong Polytechnic Normal University, Guangzhou 510665, China; pxue2008@163.com; 4 College of Artificial Intelligence, Nankai University, Tianjin 300350, China; junzhang@ieee.org; 5 Department of Electrical and Electronic Engineering, Hanyang University, Ansan 15588, Republic of Korea

**Keywords:** set packing, evolutionary algorithms, local search, approximation algorithm, performance guarantee, approximation ratio, runtime analysis

## Abstract

The set packing problem is a core NP-complete combinatorial optimization problem which aims to find the maximum collection of disjoint sets from a given collection of sets, *S*, over a ground set, *U*. Evolutionary algorithms (EAs) have been widely used as general-purpose global optimization methods and have shown promising performance for the set packing problem. While most previous studies are mainly based on experimentation, there is little theoretical investigation available in this area. In this study, we analyze the approximation performance of simplified versions of EAs, specifically the (1+1) EA, for the set packing problem from a theoretical perspective. Our analysis demonstrates that the (1+1) EA can provide an approximation guarantee in solving the *k*-set packing problem. Additionally, we construct a problem instance and prove that the (1+1) EA beats the local search algorithm on this specific instance. This proof reveals that evolutionary algorithms can have theoretical guarantees for solving NP-hard optimization problems.

## 1. Introduction

The set packing problem is a classical and significant NP-complete combinatorial optimization problem, which is included in 21 NP-complete problems famously established by Karp [1]. In the set packing problem, we are given a base set containing *m* elements, along with a collection of *n* subsets derived from the base set. The objective is to maximize the count of disjoint sets within this collection [2]. The set packing problem has attracted widespread attention from scholars in academic research and engineering fields, and has been investigated for decades. This problem has extensive practical applications, such as scheduling [3], combinatorial auctions [4], communication networks [5], product management [6], and computer vision [7], just to name a few. The significance of set packing optimization lies in its ability to efficiently allocate resources, schedule tasks, and optimize operations by selecting a subset of items while maximizing the overall value or minimizing the costs involved. Therefore, the research on the set packing problem has important application value and practical significance.

Considering the inherent complexity of the set packing problem, researchers have devoted their efforts to finding efficient approaches to address this challenging task. However, unless P=NP, it is unlikely that we can discover an algorithm for solving the set packing problem in polynomial time. Therefore, we usually only find satisfactory or approximate solutions to this NP-hard problem in practice. Hurkens and Schrijver [8] introduced a local search method that incorporates a *p*-neighborhood for solving the set packing problem. The results indicated that the local search technique can obtain $\frac{k}{2}+\epsilon$ approximate solutions on this problem for any ϵ>0. Sviridenko and Ward [9] proposed a local improvement technique that incorporates the color-coding technique, resulting in an improved approximation ratio from $\frac{k}{2}+\epsilon$ [8] to $\frac{k+2}{3}$. Cygan [10] considered local search techniques with swaps of bounded pathwidth, which resulted in a polynomial time approximation algorithm with an approximation ratio of $\frac{k+1+\epsilon }{3}$. Later, Fürer and Yu [11] achieved the same approximation ratio $\frac{k+1+\epsilon }{3}$ as [10]. However, the runtime of [11] is singly exponential in $\epsilon^{-2}$ while it is doubly exponential in $\epsilon^{-1}$ for [10]; the time complexity is decreased.

In the context of the set packing problem, when assigning weights to each subset, it is called the weighted set packing problem. Bafna et al. [12] studied a local improvement heuristic for the weighted set packing problem, resulting in a tight approximation ratio of $k-1+\frac{1}{k}$. Arkin and Hassin [13] obtained the same approximation guarantee for the problem by using a local improvement method, and extended to a general bound k−1+ϵ for any fixed ϵ. Furthermore, Chandra and Halldórsson [14] proposed a greedy strategy called BESTIMP for the *k*-set packing problem, which combines greedy with local improvement and achieved an approximation guarantee of $\frac{2(k+1)}{3}$. They also proposed another greedy strategy called ANYIMP which can find a bigger improvement than a specified threshold rather than seeking the best improvement. The ANYIMP algorithm can obtain an approximation ratio $\frac{4k+2}{5}$ for the optimal threshold selection. Recently, based on the local search procedure of Berman’s algorithm [15], Thiery and Ward [16] proposed an improved squared-weight local search algorithm with a large exchange for the weighted *k*-set packing problem. It is shown that, for the weighted *k*-set packing problem, when performing exchanges of size reaching to $2k^{2}\left(k-1\right)+k$, the algorithm can attain an approximation ratio of $\frac{k+1-\tau_{k}}{2}$ for $\tau_{k}\geq \tau_{3}=0.214$. The authors presented the analysis and design of a local search algorithm that incorporates extensive exchanges within a large neighborhood for this problem. Recently, Neuwohner [17] also studied the weighted *k*-set packing problem; some approximation guarantees are obtained by combining local search with the application of a black box algorithm. Gadekar [18] investigated the parameterized complexity on the set packing problem; he constructed a gadget called the compatible intersecting set system pair to obtain some running time bounds for the parameterized set packing problem. Moreover, Duppala et al. [19] proved that the randomized algorithms can achieve a probabilistically fair solution with provable guarantees for a fair *k*-set packing problem.

Evolutionary algorithms (EAs) [20,21,22] belong to a broad category of stochastic heuristic search methods that draw inspiration from natural processes, which can effectively solve optimization problems without any prior knowledge. Due to their simplicity and ease of implementation, EAs have been widely used to solve various complex problems, which show superior search capabilities compared to local search and greedy algorithms [23,24,25,26,27,28,29]. Generally, EAs solve optimization problems by updating the population via selection (to select the better individual to enter the next generation), crossover mutation (to generate the new offspring), and fitness evaluation (to evaluate the performance of different individuals) operators [30,31,32,33]. Since EAs have shown remarkable success in practice, the need for theoretical underpinnings is crucial to help understand the algorithm’s behavior, convergence properties, and performance guarantees. In order to further investigate the execution mechanism and essence of EAs, many scholars have carried out some theoretical research for evolutionary algorithms and obtained some achievements in the past few years. Especially He and Yao [34,35,36,37], Neumann [38,39], Qian, Yu and Zhou [40,41], and Xia et al. [42,43,44] who obtained a series of theoretical research results of evolutionary algorithms, including average convergence rate, noisy evolutionary optimization, evolutionary Pareto optimization, evolutionary discrete optimization, etc. EAs have also been widely used for solving the set packing problem. From an experimental perspective, the research findings indicate that EAs demonstrate favorable performance when applied to the set packing problem [45,46,47].

However, there has been limited research conducted on the theoretical analysis of EAs’ performance for the set packing problem. Therefore, it is natural to question whether evolutionary algorithms can obtain some performance guarantees on the set packing problem. We concentrate on the worst-case approximation guarantee achieved by some stochastic algorithms for any problem instance. That is, what the quality of solutions generated by EAs is in what expected runtime, and how the relationships between the solution quality and runtime are. This will help strengthen the theoretical foundation of EAs, and provide guidance to the design of the algorithms.

In this study, we address this question by presenting an analysis of the approximation performance of evolutionary algorithms on the set packing problem. Our primary emphasis lies in the approximation ratio achieved by the algorithms, as well as when the algorithms can obtain this approximation ratio. Specifically, we focus on a simplified variant of an EA known as (1+1) EA, which employs only the mutation operator to iteratively generate improved solutions, and the population size is one. Our investigation focuses on analyzing the performance guarantees of the (1+1) EA when it is applied to the set packing problem. Our analysis reveals that the (1+1) EA has the capability to achieve an approximate ratio of $\frac{k}{2}+\epsilon$ on the *k*-set packing problem within an expected runtime of $O(n^{2p})$, where ϵ>0 and $p=O(\log_{k}\frac{1}{\epsilon })$ is an integer. Moreover, we generate a specific instance of the *k*-set packing problem and demonstrate that the (1+1) EA can effectively discover the global optima within an expected polynomial runtime. In contrast, local search algorithms tend to become trapped in local optima when dealing with this particular instance.

The rest of this paper is structured as follows. In the subsequent section, we provide an introduction to the set packing problem, along with an overview of the relevant algorithms such as local search and greedy approaches. Additionally, we discuss the analysis tools that are considered in this study. In Section 3, we conduct a theoretical analysis of the approximation guarantees of the (1+1) EA for the *k*-set packing problem. Additionally, Section 4 provides a performance analysis for the (1+1) EA applied to a problem instance that we construct. Finally, the conclusions are presented in Section 5.

## 2. Preliminaries

This section begins by providing a comprehensive description of set packing problems and the corresponding heuristic algorithms. Moreover, we introduce pertinent concepts and analysis tools that will be utilized throughout this paper.

### 2.1. Set Packing Problem

To begin, we present the concept of an independent set before introducing the specific set packing problem addressed within this work.

**Definition** **1.**  *(Independent set) For an undirected graph G=(V,E), where V represents the set of vertices, an independent set I⊆V is defined as a subset of vertices in which no two vertices are adjacent to each other. In other words, there are no edges in E connecting any two vertices within the set I.*

The set packing problem can be considered as an extension or generalization of the independent set problem [48].

In the set packing problem, we are given a universal set of *n* elements denoted as U={1,2,⋯,N}, along with a collection $C=\{C_{1},C_{2},\cdots ,C_{n}\}$ consisting of subsets of *U*. The problem is to identify a collection $C^{\prime }\subseteq C$ of sets from *C* that are pairwise disjoint. The goal is to maximize the cardinality of $C^{\prime }$, represented as $|C^{\prime }|$.

A set packing corresponds to an independent set in the intersection graph. Hence, we give the formal definitions of the set packing problem in the context of an undirected graph.

**Definition** **2.**  *(Set packing problem) For an undirected graph G=(V,E) with a vertices set V={1,2,⋯,N}, and a collection $C=\{C_{1},C_{2},\cdots ,C_{n}\}$ consisting of subsets of V, we aim to discover a collection $C^{\prime }\subseteq C$ of sets from C that are pairwise disjoint. The goal is to maximize the cardinality of $C^{\prime }$, denoted as $|C^{\prime }|$.*

**Definition** **3.**  *(k-Set packing problem) In the set packing problem, when there is a constraint on the size of $C_{i}$ such that $\left|C_{i}\right|\;\leq \;k$ for every i∈[n], it is referred to as the k-set packing problem.*

### 2.2. Local Search Algorithm

The local search algorithm is a commonly used heuristic approach for solving the set packing problem. It operates by iteratively modifying the current solution in a local manner to improve its quality. A local search approach for the *k*-set packing problem was introduced by Hurkens and Schrijver [8]. The algorithm begins with an empty set *I* and examines whether there exist t≤p disjoint sets that are not part of the current solution set. These sets should intersect at most t−1 sets from the current collection. If such a collection *S* of *t* sets is found, the algorithm improves the current solution by adding *S* while simultaneously removing all sets from the current solution that intersect any set in *S* (up to t−1 sets). This local search algorithm is given in  Algorithm 1 below. The search continues until no further improvement can be made. The resulting solution is referred to as *p*-optimal (*p*-opt).
**Algorithm 1:** p-local search algorithm for the k-set packing problem**Input:** Graph G=(V,E);**Output:** Maximum Independent Set *I*;
1: Initialize: I←∅;
2: **While** termination condition does not hold **do**3:    Find a set $I_{good}\subseteq C\setminus I$ such that $|I_{good}|⩽p$ and the sets in $I_{good}$         are disjoint.4:    $I_{bad}=\left\{x\in I|x\cap \left(\cup I_{good}\right)\neq \emptyset \right\}$;5:    **if** $|I_{good}\left|>\right|I_{bad}|$ **then**6:       $I=I\cup I_{good}\setminus I_{bad}$;7:  **end if**8: **end while**  

As shown in Algorithm 1, we can see that the *p*-local search algorithm aims to iteratively enhance the current solution in order to achieve an improved solution.

### 2.3. Analysis Method

This section presents a fitness-level method (also known as fitness-based partitions) [49], a theoretical analysis technique. The fitness-level method is a straightforward yet useful approach for estimating the expected runtime of evolutionary algorithms and other randomized search heuristics applied to combinatorial optimization problems. Next, we provide the formal definition of the fitness-based partitions method.

**Definition** **4.**  *(Fitness-level method). Let $S={\left\{0,1\right\}}^{n}$ be a finite search space, and let f:S→R be a fitness function that needs to be maximized. For any two sets A and B that are subsets of S, we define $A{<}_{f}B$ if f(a)<f(b) holds for all a in A and all b in B. To analyze the search process, we partition the search space S into disjoint and non-empty sets $A_{1},A_{2},\cdots ,A_{m}$, satisfying the ordering*

$$A_{1}{<}_{f}A_{2}{<}_{f}\cdots {<}_{f}A_{m}.$$

*The set $A_{m}$ exclusively comprises optimal search points. We denote $p_{i}$ as the lower bound probability that, in the subsequent iteration, a new search point $x^{\prime }$ will be generated from the current search point x, where $x^{\prime }$ belongs to $A_{i+1}\cup A_{i+2}\cup \cdots \cup A_{m}$. Additionally, we define $T_{f}$ as the expected number of generations required by the (1+1) EA to discover an optimal search point for the fitness function f, then we have*

$$E\left(T_{f}\right)\leq \sum_{i=1}^{m-1}\frac{1}{p_{i}}.$$

The fitness-level method is a simple yet efficient approach for runtime analysis for EAs, also called fitness-based partitions. However, this technique only presents the upper bound estimation, and a general lower bound estimation method is difficult to establish. It should be pointed out that if you use this method, you have to give an appropriate partition for the search space *S*. The number of partitions cannot be too large, i.e., exponential. Furthermore, it is relatively straightforward to estimate the probability of transitioning from the current partition to a better partition and generating a new offspring.

## 3. Theoretical Analysis of a Simple Evolutionary Algorithm for k-Set Packing Problem

In this section, we focus on analyzing the approximation guarantee of evolutionary algorithms applied to the set packing problem. An approximation algorithm is designed to provide a solution for a combinatorial optimization problem, such that its objective value is guaranteed to be within a bounded factor of the optimal solution’s value. The approximation ratio serves as a fundamental metric for evaluating the quality of an approximation algorithm.

Let us begin by introducing the notion of approximation ratio. Without a loss of generality, we consider a maximization problem with an objective function denoted as *f*. For any given instance *I*, the value of an optimal solution is represented by OPT(I). An algorithm is considered a ρ-approximation algorithm if the best solution *x* obtained by the algorithm satisfies $f\left(x\right)\geq \frac{1}{\rho }\cdot OPT\left(I\right)$, where ρ≥1. In other words, the algorithm achieves a solution with a value that is at least $\frac{1}{\rho }$ times the value of the optimal solution. We refer to this as the ρ-approximation ratio for the problem. If the value of ρ is equal to 1, it indicates that the algorithm is considered optimal. It is worth noting that a higher approximation ratio indicates a worse solution in terms of quality.

Next, we present the (1+1) EA used in this study as shown in Algorithm 2.
**Algorithm 2:** The (1+1) EA for the set packing problem**Input:** A Graph G=(V,E);
**Output:** A vertices set *C* with the cardinality of *C* as maximized as possible.
1: Initialization: Randomly select an initial bit string $x\in {\left\{0,1\right\}}^{m}$;2: **While** termination condition is not met **do**3:    Generate an offspring $x^{\prime }$ by independently flipping each bit of *x* with         a probability of 1/n;4:    **if** $f\left(x^{\prime }\right)>f\left(x\right)$ **then**5:      $x:=x^{\prime }$.6:   **end if**7: **end while**  

The (1+1) EA begins with an arbitrary solution and proceeds iteratively by generating an offspring solution using the mutation operator but without crossover. The current solution is replaced by the new solution only if it is strictly superior to the current one.

To utilize the (1+1) EA for finding the optimal or approximate optimal solution to the *k*-set packing problem, we employ a bit string $x=\left(x_{1},x_{2},\cdots ,x_{n}\right)\in {\left\{0,1\right\}}^{n}$ to encode the problem’s solution. Each bit $x_{i}$ corresponds to a subset $C_{i}$, where i∈[n]. If $x_{i}=1$, it indicates that the subset $C_{i}$ is selected. Conversely, if $x_{i}=0$, it implies that $C_{i}$ is not selected. Consequently, a solution or bit string *x* represents a collection of subsets, and $\left|x\right|=\sum_{i=1}^{n}x_{i}$ represents the count of subsets contained in *x*.

In the following, we define the fitness function for the *k*-set packing problem, denoted as

(1) $$f\left(x\right)=\left|x\right|-n^{2}\times u\left(x\right).$$

We aim to maximize the fitness function f(x). In the above Equation (1), the item u(x) represents the number of subsets with an intersection. The penalty term in the fitness function, $n^{2}\times u\left(x\right)$, serves to encourage the algorithm to minimize the value of u(x). This, in turn, leads to a reduction in the number of intersecting subsets within the current solution. The primary goal is to obtain a solution where all subsets $C_{i}$ are disjoint. The first part of Equation (1) ensures that the number of disjoint subsets is maximized if all subsets are disjoint.

Hurkens and Schrijver [8] demonstrated that a simple local search algorithm can attain a polynomial time approximation ratio on the *k*-set packing problem.

**Lemma** **1**  ([8]). *For any ϵ>0, let p be an integer and $p=O(\log_{k}\frac{1}{\epsilon })$. The local search algorithm with p−local improvement can achieve an approximation ratio $\frac{k}{2}+\epsilon$ when applied to any instance of the k-set packing problem.*

Next, we demonstrate that the (1+1) EA can effectively discover a $\frac{2}{k+2\epsilon }$-approximation solution for the unweighted *k*-set packing problem, while simulating the aforementioned result established by Hurkens and Schrijver [8]. Additionally, the expected runtime for this achievement is bounded by $O(n^{2p})$.

The (1+1) EA has the capability to discover a packing that includes a minimum number of sets, reaching at least $\frac{2}{k+2\epsilon }OPT$, for the *k*-set packing problem. This accomplishment is expected to occur within a runtime of $O(n^{2p})$, where OPT represents the global optimum.

**Theorem** **1.**  *The (1+1) EA has the capability to discover a packing with a number of sets that is at least $\frac{2}{k+2\epsilon }OPT$ for the k-set packing problem. This achievement can be accomplished within an expected runtime of $O(n^{2p})$, where OPT represents the global optimum.*

**Proof.**  During the optimization process of the (1+1) EA, it is important to note that the value of the fitness function is never reduced. To facilitate this, the search space ${\left\{0,1\right\}}^{n}$ is divided into three distinct sets, namely $P_{1}$, $P_{2}$, and $P_{3}$, based on their respective fitness values.

$$P_{1}=\left\{x|x\in {\left\{0,1\right\}}^{n},f\left(x\right)<0\right\},$$

$$\begin{array}{cc} P_{2}= & \{x|x\in {\left\{0,1\right\}}^{n},0\leq f\left(x\right)<\frac{2}{k+2\epsilon }OPT\}, \end{array}$$

$$\begin{array}{cc} P_{3}= & \{x|x\in {\left\{0,1\right\}}^{n},f\left(x\right)\geq \frac{2}{k+2\epsilon }OPT\}. \end{array}$$

It is obvious that $P_{1}$ represents the set of infeasible solutions, and $P_{2}$ and $P_{3}$ represent the set of feasible solutions.

In accordance with the fitness function (1), the fitness values in the evolutionary process of the (1+1) EA are guaranteed to never decrease. The (1+1) EA initiates by randomly selecting an initial solution from the search space. Suppose the current solution *x* belongs to $P_{1}$, then *x* is a collection of subsets containing intersection, i.e., an infeasible solution. At least two subsets in the current solution are intersecting. The algorithm only accepts the event that reduces the number of subsets with an intersection. In this scenario, the (1+1) EA has the capability to enhance the fitness value by at least one by removing such a subset with an intersection from the solution. In this case, the probability of removing such a specific subset is $\frac{1}{n}{\left(1-\frac{1}{n}\right)}^{n-1}\geq \frac{1}{en}=\Omega \left(\frac{1}{n}\right)$; this suggests that the fitness value is expected to increase by at least one within a runtime of O(n). Note that the maximum fitness value is *n*. Consequently, the solutions from set $P_{1}$ will be transformed into set $P_{2}$ by the (1+1) EA, and this transformation will be accomplished within an expected runtime of $O(n^{2})$.

Let us assume that the current solution *x* belongs to set $P_{2}$. On the basis of Lemma 1, if some solution satisfies $0\leq f\left(x\right)<\frac{2}{k+2\epsilon }OPT$, then the algorithm evolves towards increasing the number of disjoint subsets. For this case, the following possibilities exist. There exist *p* disjoint sets that are not included in the current solution, and these *p* subsets intersect at most p−1 subsets in the current solution. Consequently, by performing a *p*-local improvement operation, the fitness value is increased by at least 1 by the (1+1) EA. The probability of performing such operation for the (1+1) EA is ${\left(\frac{1}{n}\right)}^{2p-1}{\left(1-\frac{1}{n}\right)}^{n-2p+1}\geq \frac{1}{en^{2p-1}}=\Omega \left(\frac{1}{en^{2p-1}}\right)$. Since there are most *n* elements in the solution, according to the fitness-level method, performing such *n* operations, the (1+1) EA will transform the solutions into set $P_{3}$ within an expected runtime of $O(\frac{1}{n^{2p}})$.

Combining the above analysis for different phases, we complete the proof. □

As shown in Theorem 1, the (1+1) EA can achieve a performance guarantee equivalent to that of the local search algorithm in the worst case. Understanding the theoretical properties of different algorithms can guide researchers and practitioners in choosing the most suitable approach based on the problem characteristics. Applying theoretical guarantees to real-world optimization problems can help in predicting algorithm behavior and performance outcomes. By leveraging theoretical insights, practitioners can tailor optimization algorithms to specific problem domains and optimize them for practical applications.

## 4. The Power of Evolutionary Algorithms on Some Instances of the k-Set Packing Problem

Local search and greedy algorithms are widely used and are practical approaches for efficiently searching approximate solutions in various optimization problems. However, both of these techniques are prone to becoming stuck in local optima. In this section, we present a constructed instance of the *k*-set packing problem. We demonstrate that the simple evolutionary algorithm has the capability to discover global optimal solutions within an expected polynomial runtime. On the other hand, the 2-local search algorithm may be apt to fall into the local optimum.

First, we give two sets with base elements as follows.

$$A=\{v_{1},v_{2},\cdots ,v_{s}\},$$

and

$$\begin{array}{cc} B= & \{u_{1},u_{2},\cdots ,u_{s},u_{1,2},\cdots ,u_{1,s},u_{2,3},\cdots ,u_{2,s},\\ \; & \cdots ,u_{i,i+1},u_{i,i+2},\cdots ,u_{i,s},\cdots ,u_{s-1,s}\}, \end{array}$$

where *s* is an integer.

The instance $I_{sp}$ is constructed using the following steps. First, we construct three groups of sets $C_{i,0}$, $C_{0,i}$ and $C_{i,j}$. Let

$$\begin{array}{cc} C_{i,0}= & \{\left\{v_{i},u_{i}\right\},\left\{v_{i},u_{1,i}\right\},\cdots ,\left\{v_{i},u_{i-1,i}\right\},\left\{v_{i},u_{i,i+1}\right\},\\ \; & \cdots ,\{v_{i},u_{i,s}\}\},\;where\;2\leq i\leq s-1. \end{array}$$

Here, $C_{1,0}=\left\{\left\{v_{1},u_{1}\right\},\left\{v_{1},u_{1,2}\right\},\cdots ,\left\{v_{1},u_{1,s}\right\}\right\}$, and $C_{s,0}=\{\left\{v_{s},u_{s}\right\},\left\{v_{s},u_{1,s}\right\},$$\left\{v_{s},u_{2,s}\right\},\cdots ,\left\{v_{s},u_{s-1,s}\right\}\}$.

If the subscript of *u* is 0, it means that the element does not exist.

For 1≤i≤s, we set

$$C_{0,i}=\left\{\left\{v_{i},u_{i}\right\}\right\}$$

and

$$C_{i,j}=\left\{\left\{v_{i},u_{i,j}\right\},\left\{v_{j},u_{i,j}\right\}\right\}.$$

**Figure 1:**
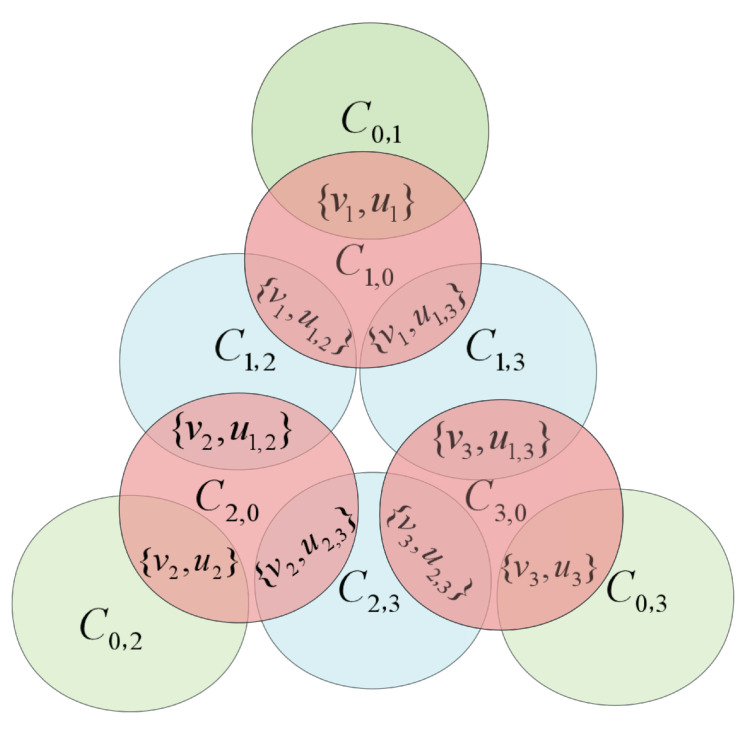
Instance $I_{sp}$ with s=3.

After that, we define two sets *X* and *Y*. For any 1≤i≤s, let $X=\{C_{1,0},C_{2,0},\cdots ,C_{s,0}\}$, $Y_{1}=\left\{C_{0,1},C_{0,2},\cdots ,C_{0,s}\right\}$ and $Y_{2}=\left\{C_{1,2},\cdots ,C_{1,s},C_{2,3},\cdots ,C_{2,s},\cdots ,C_{s-1,s}\right\}$. The set $Y=Y_{1}⋃Y_{2}$. We can see that the size of set *X* is *s*, i.e., |X|=s, and $\left|Y|=\right|Y_{1}\left|+\right|Y_{2}|=s+\frac{s(s-1)}{2}$. The number of all elements contained in sets *X* and *Y* is *n*. The constructed instance $I_{sp}$ is shown in Figure 1.

In the following analysis, we demonstrate that the (1+1) EA can achieve a global optima for instance $I_{sp}$ within an expected polynomial runtime.

**Theorem** **2.**  *For instance $I_{sp}$, the (1+1) EA can efficiently obtain the global optimum starting from any initial solution within an expected runtime of $O(n^{5})$.*

**Figure 2:**
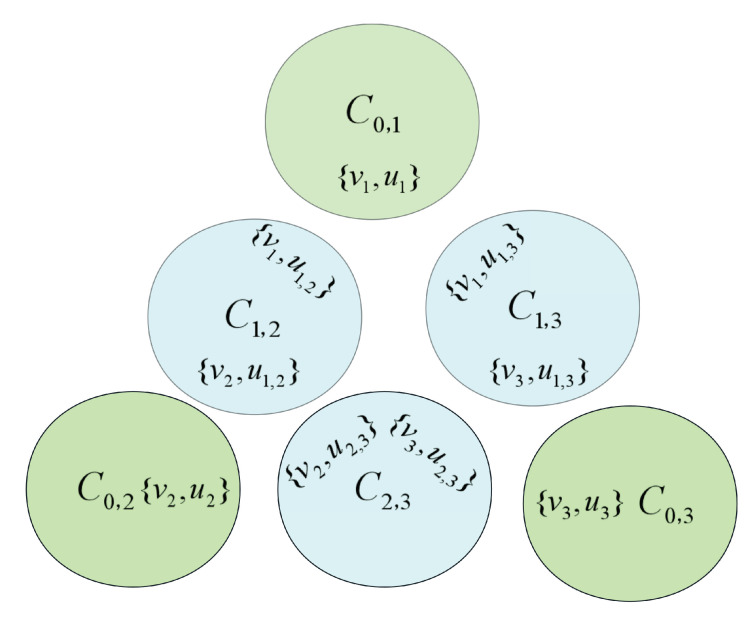
The global optimum of $I_{sp}$ with s=3.

**Proof.**  Obviously, the global optimum of $I_{sp}$ is $\left\{C_{0,1},\cdots ,C_{0,s},C_{1,2},\cdots ,C_{1,s},C_{2,3},\cdots ,C_{2,s},\cdots ,C_{s-1,s}\right\}$ as shown in Figure 2. We can divide the optimization process of the algorithm on $I_{sp}$ into two stages. In the first stage, the fitness value is strictly negative, while in the second stage, the fitness value becomes positive.

In the first stage, since the fitness value is strictly less than 0, the intersection of some subsets exists in the current solution. In this case, the (1+1) EA will only accept operations that reduce the number of intersections in the subset. The probability that the algorithm reduces a particular subset with intersection is $\frac{1}{n}{\left(1-\frac{1}{n}\right)}^{n-1}=\Omega \left(\frac{1}{n}\right)$. It is noticed that the total set contains *n* elements. By employing the fitness-level method, the (1+1) EA is transitioned from the first stage to the second stage within an expected runtime of $O(n^{2})$.

During the second stage, the subsets within the current solution are mutually disjoint. It is necessary to consider four distinct cases in this context.

**Figure 3:**
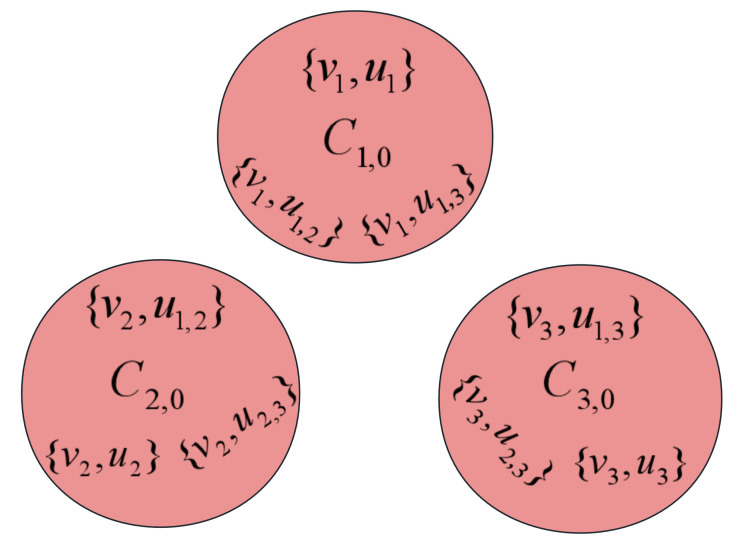
A 2-optimum of $I_{sp}$ with s=3.

Case 1: All the elements from *X* are included in the current solution, i.e., a 2-optimal solution, as shown in Figure 3. In this case, any subset in *Y* can not be included into the current solution. Because the algorithm only accepts better solutions, this case occurs only once during the optimization process. We claim that the fitness value can be increased by at least one by the (1+1) EA through deleting two subsets $C_{i,0}$ and $C_{j,0}$ in *X* and simultaneously adding three subsets $C_{0,i}$, $C_{0,j}$ and $C_{i,j}$ in *Y*. In the following, we estimate the probability that the algorithm performs this operation. We first choose one element from the set $Y_{2}$, where the probability for this operation is $\frac{s(s-1)}{2n}$. We assume that we choose the element $C_{i,j}$, then we need to remove the elements $C_{i,0}$ and $C_{j,0}$ from set *X* while adding the elements $C_{0,i}$ and $C_{0,j}$ from set *Y* simultaneously. Such an improvement will occur with probability $\frac{s(s-1)}{2n}\frac{1}{n^{4}}{\left(1-\frac{1}{n}\right)}^{n-5}=\Omega \left(\frac{1}{n^{4}}\right)$, which implies that the fitness value can be increased by at least one in the expected runtime $O(n^{4})$.

Case 2: The current solution contains, at most, s−1 elements selected from *X*, but there are no elements from *Y* contained in the current solution. In this scenario, by either adding the elements from set *X* or removing the elements from set *Y* that are disconnected from the elements in set *X*, the fitness value can be increased. Such an operation is executed by the (1+1) EA with probability $\frac{1}{n}{\left(1-\frac{1}{n}\right)}^{n-1}=\Omega \left(\frac{1}{n}\right)$, which implies that the fitness value can be increased by at least one in expected runtime O(n).

Case 3: The current solution contains at most s−1 elements selected from *X*, also there exist some elements from *Y* contained in the current solution. For this case, we can divide it into two subcases for further consideration.

Subcase 3.1: The current solution contains less than s−1 elements selected from *X*. Let us assume that the subset $C_{0,k}$ is included in the current solution but without $C_{i,0}$ and $C_{j,0}$ for the sake of simplicity. Based on the aforementioned analysis, we can conclude that the current solution forms an independent set. Therefore, neither subset $C_{i,k}$ nor $C_{j,k}$ is present in the current solution. Through adding subsets $C_{i,k}$ and $C_{j,k}$ while simultaneously removing subset $C_{0,k}$ from set *X* by the (1+1) EA, the fitness value will be increased. Such an operation is executed by the (1+1) EA with probability $\frac{1}{n^{3}}{\left(1-\frac{1}{n}\right)}^{n-3}=\Omega \left(\frac{1}{n^{3}}\right)$, which implies that the fitness value can be increased by at least one in the expected runtime $O(n^{3})$.

Subcase 3.2: The current solution contains exactly s−1 elements from *X*, meaning that only a single element in *X* is excluded form the current solution. For the sake of simplicity, let us assume that the element $C_{i,0}$ from *X* is not included in the current solution. Furthermore, since the current solution is an independent set, we can know that only the element $C_{0,i}$ in *Y* is contained in the current solution. Through adding two subsets $C_{0,j}$ and $C_{i,j}$ and simultaneously deleting the subset $C_{j,0}$ from *X* by the (1+1) EA, the fitness value will be increased. The probability of the (1+1) EA executing such an operation is $\frac{1}{n^{3}}{\left(1-\frac{1}{n}\right)}^{n-3}=\Omega \left(\frac{1}{n^{3}}\right)$, which implies that the fitness value can be increased by at least one in the expected runtime $O(n^{3})$.

Case 4: The current solution exclusively consists of the elements from set *Y*. For this case, through adding any other element from *Y* into the current solution by the (1+1) EA, the fitness value can be increased by at least one. Such an operation is executed by the (1+1) EA with probability $\frac{1}{n}{\left(1-\frac{1}{n}\right)}^{n-1}=\Omega \left(\frac{1}{n}\right)$, which implies that the fitness value can be increased by at least one in the expected runtime O(n).

Note that there are mostly *n* elements in the solution, i.e., the maximum fitness value is *n*. Therefore, according to the above analysis, starting with an arbitrary solution, the (1+1) EA can obtain the global optimal solution of instance $I_{sp}$ in an expected runtime of $O(n^{5})$. □

As we can see from Theorem 2, as a global optimization method, the evolutionary algorithm can avoid becoming stuck in local optima, but it is easy for the local search algorithm to become trapped in local optima.

## 5. Conclusions and Future Work

EAs have been commonly used for tackling the set packing problem, an NP-complete combinatorial optimization problem, and some experimental investigations have demonstrated that EAs exhibit efficiency in solving this problem. However, the understanding of the performance of EAs in the set packing problem remains limited in theory. This work focuses on the performance analysis of a simple but effective (1+1) EA on the set packing problem. We have demonstrated that the simple algorithm can obtain some approximation guarantees on the set packing problem. Furthermore, we theoretically reveal that the (1+1) EA outperforms the local search algorithm on a set packing instance. This study contributes to bridging the gap between theory and practice in the context of evolutionary algorithms. Theoretical investigation can give insight in how algorithms work and help select the most appropriate algorithm for a given problem. By understanding these theoretical insights into practical scenarios, researchers and practitioners can make decisions, optimize algorithm performance, and enhance the reliability of algorithms across practical applications.

In the future, we will further conduct other theoretical analysis for the more complex EAs on the *k*-set packing problem, and will also consider other theoretical analysis methods, such as parameter analysis, in some practical intelligence optimization algorithms [50,51,52,53] to see whether EAs can still maintain the approximation guarantees, or potentially improve the existing approximation guarantees established in the set packing problem. We can study the impact of crossover operators on algorithm performance. It is also interesting to theoretically analyze the approximation performance on the weighted *k*-set packing problem [54]. This will help to provide some valuable insights into the correlation between problem features and algorithm performance, facilitating a deeper understanding of their relationship. Evolutionary algorithms are commonly used to solve complex optimization problems in various fields, they are expected to provide theoretical evidence for more applications, e.g., machine learning and multi-objective optimization.

## Data Availability

The data that support the findings of this study are available from the corresponding author upon request. There are no restrictions on data availability.
